# Increasing Fruit and Vegetable Consumption Among Preschool-Age Children and Their Families: Protocol for the Project V.E.G.G.I.E. Pilot/Feasibility Study

**DOI:** 10.2196/93866

**Published:** 2026-11-13

**Authors:** Anne-Charles Zimmer, Jhada Sims, Keith Brazendale, Amy R Mobley, JeeWon Cheong, Layton Reesor-Oyer

**Affiliations:** 1 Department of Health Education and Behavior, College of Health and Human Performance, University of Florida, P.O. Box 118210, Gainesville, FL, 32611, United States, 1 (352) 294-2712; 2 Department of Health Sciences, University of Central Florida, Orlando, FL, United States

**Keywords:** food insecurity, fruit and vegetable consumption, parent feeding practices, nonrandomized trial, child eating behaviors

## Abstract

**Background:**

Preschool-age children (2‐5 y) living in households experiencing food insecurity are at a higher risk of facing health and behavioral issues, as well as consuming lower-quality diets with fewer fruits and vegetables (FVs). Repeated exposures are necessary for children to accept certain commonly rejected/disliked foods, but parents in households experiencing food insecurity may not purchase foods their child does not readily accept. Project V.E.G.G.I.E. (Vegetable Eating Gets Going by Increasing Exposure) aims to address this issue by providing families with FVs at no cost, alongside education on evidence-based parent-feeding practices tailored to the needs of preschool-aged children.

**Objective:**

This study describes the protocol for the Project V.E.G.G.I.E. pilot/feasibility study.

**Methods:**

The cluster nonrandomized controlled trial includes 20 dyads: parents and their preschool-age children. Families received 6 boxes of fresh FVs biweekly for 10 weeks, alongside educational materials on parent feeding practices. Parent surveys and daily diaries were completed at 3 time points: baseline, posttest (weeks 10‐11), and follow-up (4 wk after intervention). Participants in the intervention group also completed an exit survey to assess the acceptability and utility of the FV boxes across several domains, including quantity, quality, and variety. Standard effect size estimates (Cohen *d*) will be calculated as baseline to posttest and baseline to follow-up analyses. Lastly, both control and intervention participants were invited to complete a qualitative interview to discuss satisfaction with the program.

**Results:**

The Project V.E.G.G.I.E. program was funded internally by the Department of Health Education and Behavior at the University of Florida. Recruitment began in February 2025, and data collection took place from March to July 2025. Data cleaning is underway at the time of submission (February 2026); we expect to submit the outcome/feasibility manuscript in fall 2026.

**Conclusions:**

The results of the Project V.E.G.G.I.E. study will be used to evaluate the feasibility and acceptability of this pilot intervention. Feedback will inform refinement of the Project V.E.G.G.I.E. intervention and study protocol prior to scaling up to a well-powered cluster randomized controlled trial. Implementing programs like Project V.E.G.G.I.E. that provide families with increased access to FVs at childcare settings may overcome barriers typically associated with FV initiatives. Providing families with both increased access to FVs and education on parent-feeding practices may be more effective at increasing FV consumption among preschoolers than either approach implemented independently.

## Introduction

### Background

Food insecurity (FI), the inability to acquire enough food to support a healthy, active lifestyle [1], is a major health concern in the United States [2]. Recent prevalence estimates indicate that 17.9% of households with children experienced FI at some point in 2023 [1]. This is problematic as FI is related to poor mental (depression [3], anxiety [4]) and physical (obesity [5], diabetes [6]) health outcomes among adults. Children living in households experiencing FI are at an increased risk of behavioral, academic, and socioemotional problems [7] as well as poor physical health [8]. Further, individuals experiencing FI report lower diet quality than those classified as food secure. Adults living in households experiencing FI consume fewer fruits and vegetables (FVs) [9] and a higher amount of energy-dense foods with added sugars and high-fat dairy products [10]. Children in these same households have similar dietary patterns, consuming diets high in calories, sugar, and fat [11] and low in FVs [12], compared to their food-secure peers.

Within the context of FI, nutrition security refers to the healthfulness or nutritional adequacy of available foods [13]. FV consumption is one aspect of nutrition security as it is known to be relevant for the prevention of chronic disease [14,15]. Disparities in FV consumption among those experiencing FI exist. Among children aged 1 to 5 years living in households experiencing FI, only 41% ate a daily vegetable and 54% ate a daily fruit compared with those living in food-secure households (53% vegetable; 70% fruit) [16].

An underexplored reason for disparities in FV consumption among this age group is the importance of repeated exposure to novel/disliked foods (such as vegetables). Repeated exposure is consistently identified as necessary for children to accept a variety of foods; children may need up to 15 exposures before they “like” a new food [17]. However, it is common for parents to only provide children with a novel food 3 to 5 times before determining that their child does not like it [18] and no longer offering it. Lack of repeated exposure to novel foods may be especially problematic for households experiencing FI because these families cannot afford to endure costs associated with wasted food [19]. As such, families experiencing FI may rely on highly palatable foods that they know their children will accept, leading to the disparities in FV consumption. The importance of repeated exposure to novel/disliked food is highly relevant for preschool-age (2‐5 years old) children because this developmental period often coincides with an increase in food neophobia (fear of new foods) and picky eating. The onset of picky eating typically peaks around 2 years of age and persists until age 4 to 6 years [20,21], thus creating a vulnerable developmental period when parents may decrease offering foods their child is likely to reject [19].

Children are not only influenced by their parents’ feeding practices, but also the environment around them. Many families experiencing FI may send their children to home-based childcare settings as they are normally more affordable and flexible for parents [22]. These home-based childcare providers may be in smaller settings with fewer children, focusing on a family-like environment [22]. In these settings, children also lack exposure to new FVs as the families attending may not be able to afford that food waste in or out of the house.

One strategy to increase FV consumption among families experiencing FI is to provide them with access to FVs at no cost. Such support may enable parents to provide their preschool-age children with the repeated exposures necessary for them to learn to like and eat FVs. However, the efficacy of these initiatives is mixed [23,24]. One highly relevant study which provided discounted FVs detected changes in parents’ but not children’s diets [25], indicating that access alone may not be enough to influence children’s diets.

Many programs to increase FV access involve barriers to participation such as advanced purchasing/ordering and inconvenient pick-up times/locations [25]. We attempt to address this issue by delivering FVs to childcare providers, a convenient location for parents. Families can pick up their FVs when they pick up their child. Much of the literature leveraging childcare settings has focused on center-based care providers and failed to include home-based childcare settings [26]. However, many families with low resources may not work typical hours and use home-based childcare providers due to scheduling flexibility; they are the most common form of nonparental care for young children in the United States [27]. These providers have fewer resources than those in center-based childcare settings [27], making home-based childcare providers an ideal and underutilized location to provide resources to families with preschool-age children at risk of experiencing FI.

Providing parents with evidence-based strategies to improve their own feeding practices for their child(ren) alongside increased access to FVs may be necessary to impact children’s FV consumption. In line with the Family Systems Theory [28], educating parents on evidence-based parent feeding practices may enable them to endure children’s resistance when offered novel or disliked FVs and ultimately lead to greater child FV consumption. Parents are considered “nutritional gatekeepers” for their children because they have such an important influence on children’s diets. Parents determine not just what children eat, but also when, where, and how much food children eat [29]. Such education may be particularly relevant for families experiencing FI as FI may have detrimental impacts on parent feeding practices. Parents experiencing FI may restrict access to food when resources are scarce and pressure children to eat in the absence of hunger when food is available [30]. These practices disrupt children’s innate ability to self-regulate the amount they eat; they are related to problematic eating behaviors and obesity [30]. The Satter Division of Responsibility in Feeding is considered the gold standard for evidence-based parent feeding practices. According to this framework, both the parent and child play important roles within a healthy eating environment [31]. Parents are responsible for what food is served as well as the timing and location of meals and snacks; the child is responsible for how much (and whether) they eat from the options provided by the parent [31]. Unfortunately, when a family deviates from these responsibilities (such as the child determining what is served, or the parent determining how much the child must eat), unhealthy child eating behaviors, like picky eating, may occur [31]. In addition to the distinct roles of both parent and child, the Satter Division of Responsibility in Feeding recommends the following strategies to promote a healthy diet for children: repeated exposure to novel/rejected foods, structured meals and snacks with consistent rules/routines, and the importance of parental modeling of desired eating behaviors (eg, FV consumption) [31]. When these strategies are consistently implemented, child food preferences and consumption can be improved. However, families with low resources, such as those experiencing FI, may struggle to implement such education without additional material support. Therefore, to increase FV consumption, it is necessary to provide families with preschool-age children experiencing FI with both education on evidence-based parent feeding practices as well as additional access to FVs.

### Objective

The primary objective of Project V.E.G.G.I.E. (Vegetable Eating Gets Going by Increasing Exposure) is to increase FV consumption among preschool-age children and their families at risk of experiencing FI. To achieve these goals, we propose providing families with increased access to FVs alongside education on evidence-based parent feeding practices (based on the Satter Division of Responsibility in Feeding). The goal of this study is to determine the feasibility and acceptability of the study protocol. Preliminary data will inform the format and contents of future iterations of the Project V.E.G.G.I.E. intervention as well as demonstrate feasibility, acceptability, and a preliminary signal of program efficacy. Figure 1 depicts the Project V.E.G.G.I.E. conceptual model and intervention targets.

**Figure 1.**
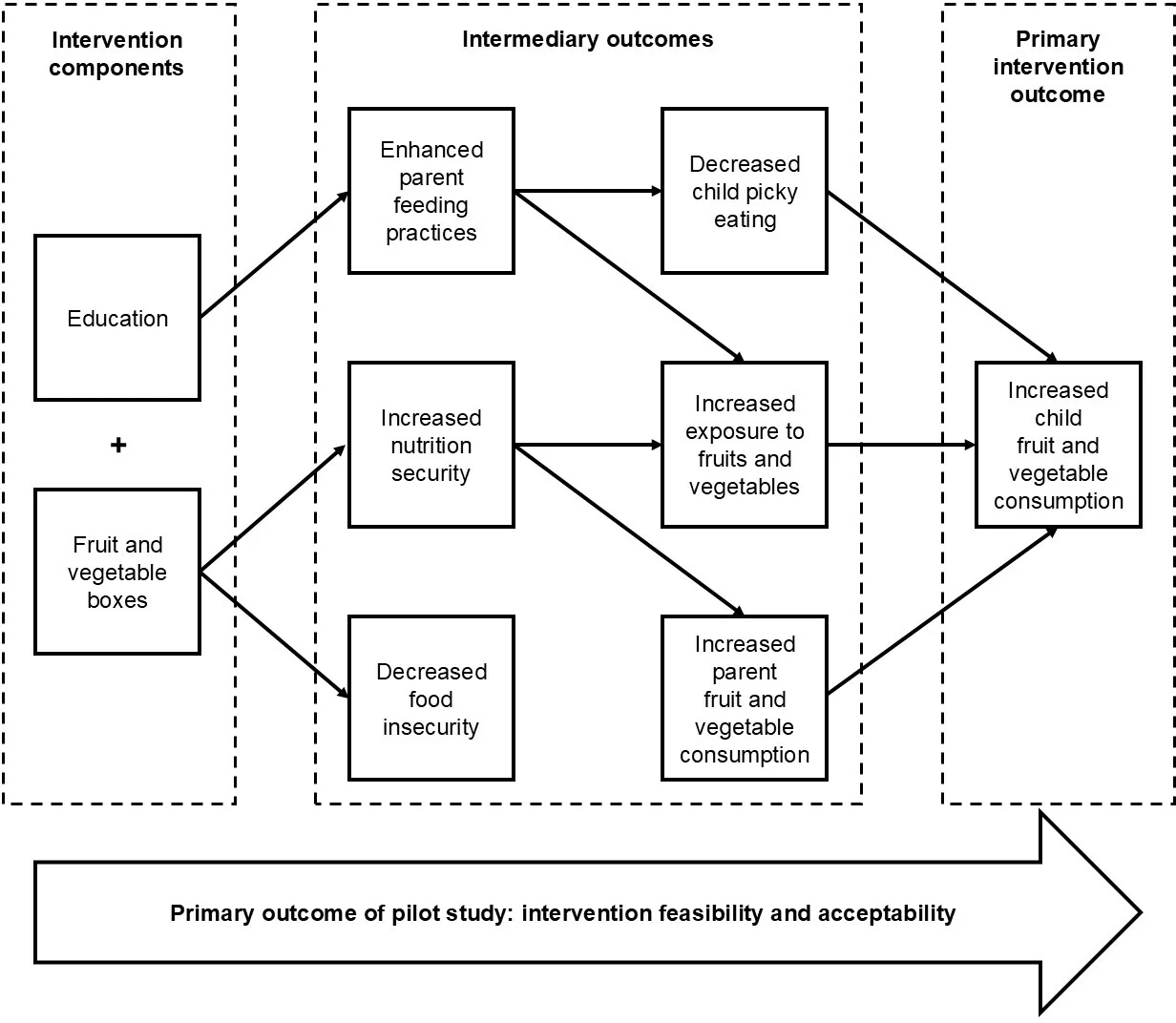
Conceptual model of Project V.E.G.G.I.E. (Vegetable Eating Gets Going by Increasing Exposure) intervention.

## Methods

### Design Overview

The Project V.E.G.G.I.E. Pilot/Feasibility Study was a cluster nonrandomized controlled trial (non-RCT). We originally planned for it to be a proof-of-concept study where all participants received the intervention. However, due to changes in the federal administration and potential changes in policy due to Department of Government Efficiency cuts [32], a control group was added to account for potential confounding variables (eg, potential cuts to social safety net programs such as the Supplemental Nutrition Assistance Program, the Special Supplemental Nutrition Program for Women, Infants, and Children, and childcare subsidies). Participants in both conditions completed assessments at 3 time points: baseline (prior to the start of the program), posttest (weeks 10‐11), and follow-up (4 weeks after the final box delivery). See Table 1 for the assessment schedule.

**Table 1.** Timeline of assessments and intervention activities.

	Project V.E.G.G.I.E.^a^	Control
Assessment/activity	Week(s) of	Week(s) of
Recruitment	February 17, 2025, to March 3, 2025	March 3, 2025, to March 10, 2025
Baseline parent survey	February 24, 2025	March 10, 2025
Baseline daily diaries	February 24, 2025	March 24, 2025
Intervention (boxes delivered)	March 3, 2025, to May 5, 2025	N/A^b^
Posttest parent survey	May 5, 2025	May 5, 2025
Posttest daily diaries	May 5, 2025	May 5, 2025
Exit survey	May 5, 2025	N/A
Exit interviews	May 12, 2025, to June 16, 2025	July 7, 2025
No contact follow-up period	May 19, 2025, to May 26, 2025	May 19, 2025, to May 26, 2025
Follow-up parent survey	June 2, 2025	June 2, 2025
Follow-up daily diaries	June 2, 2025	June 2, 2025

a V.E.G.G.I.E.: Vegetable Eating Gets Going by Increasing Exposure.

b N/A: not applicable.

### Ethical Considerations

The study protocol was approved by the institutional review board of the University of Florida (study ID: IRB202401344).

### Participants

#### Recruitment of Childcare Providers

Parents and their preschool-age children were recruited from childcare providers (N=5) located in Orange County, Florida, in February/March 2025. The research team partnered with the Early Learning Coalition of Orange County (ELCOC) to identify partner childcare providers. The ELCOC’s mission is to provide all children in the Orlando area with fair access to quality learning environments. Childcare providers in the ELCOC network are Supplemental Nutrition Assistance Program eligible and accept childcare vouchers. ELCOC staff invited childcare providers they had successfully collaborated with on previous initiatives (eg, art programs) to participate in Project V.E.G.G.I.E. All contacted childcare providers agreed to participate in the research study (n=5, 100%). Originally, 3 childcare providers (n=2 home-based; n=1 center-based) were contacted to participate in the Project V.E.G.G.I.E. Pilot/Feasibility Study, with all participants receiving the program (proof-of-concept study). An additional 2 home-based childcare providers were later invited to serve as control sites due to concerns that potential changes in federal policy could impact key intervention outcomes (eg, food/nutrition security). Because of this, recruitment of control participants and their baseline assessments occurred a few weeks later. Interested individuals were informed of group allocation during informed consent.

#### Recruitment of Participants

Partner childcare providers shared the opportunity to participate in the research study with families enrolled at their site via study flyers (hard copies) featuring a QR code that linked to the informational website, which included informed consent documents and an online screener. Additionally, childcare providers directly shared the link to the study website via their normal communication methods (apps). The recruitment process for the control and intervention participants was identical; both groups were informed of their group allocation (intervention vs control) based on their childcare provider during the online informed consent process. Participants were informed of their eligibility/enrollment status and next steps by the research team (not the childcare providers) via text message.

#### Eligibility

Eligibility criteria for primary caregivers included being at least 18 years of age and the primary caregiver (at least 50% custody) of a 2- to 5-year-old child enrolled at a childcare provider participating in the research study. Primary caregivers had to be able and willing to complete surveys in either English or Spanish with smartphone access (to complete study assessments). Exclusion criteria for primary caregivers included not having a child within the target age range (eg, <2 or >5 years old) and having a child with a medical condition that significantly impacts their diet (parent reported).

### Procedures

#### Study Timeline and Data Collection Overview

Parents completed assessments at 3 time points: baseline (prior to the start of the program), posttest (weeks 10‐11), and follow-up (4 wk after the end of the program). Parent surveys and daily diaries were distributed via text message with personalized links and completed by parents on their smartphones.

#### Intervention

The intervention group received the Project V.E.G.G.I.E. program, which included 6 boxes with 10 to 14 pounds of culturally familiar FVs biweekly delivered to their childcare provider for 10 weeks. Due to the university spring break schedule and corresponding lack of availability of undergraduate research assistants, 2 FV boxes were delivered in 2 consecutive weeks. During this time, parents also received education on evidence-based parent feeding practices.

#### FV Boxes

We partnered with Second Harvest Food Bank of Orange County to provide the FV boxes using their existing infrastructure. Undergraduate research assistants collected the FV boxes from Second Harvest and delivered them to the childcare providers. Research assistants remained at the childcare providers to facilitate distribution and collect process evaluation data (eg, number of boxes distributed). To reduce stigmatization, all families enrolled at intervention childcare providers received an FV box during each distribution, regardless of study enrollment or eligibility.

#### Educational Materials

##### Parent

Educational materials were provided in both English and Spanish. They focused on evidence-based parent feeding practices and included strategies to increase children’s FV consumption tailored to the unique needs of preschool-age children based on the Satter Division of Responsibility in Feeding [31]. Educational materials covered the following topics: normalizing child eating behaviors that are often frustrating for parents (such as inconsistent eating volume, refusing previously liked foods, and preferring snacks over meals), the importance of repeated exposure to FVs and role modeling, not pressuring children to eat certain foods, the distinct roles of parents and children at mealtimes (according to the Satter Division of Responsibility in Feeding), and noneating exposure to foods such as getting their child involved in food preparation. Based on findings from a needs assessment, education was delivered through print materials included with the FV boxes, as well as text messages (infographics) sent to parents’ smartphones.

##### Child

As an additional method of exposure [33], children also received print materials with activities and simple information about FVs. Children’s materials included topics like how various fruits and vegetables grow (carrots in the ground, apples in trees) and what animals eat vegetables, as well as a checklist to mark what FVs they received in their box that week. Children’s materials were not a primary intervention target; they were added to the research study because they were specifically requested by the ELCOC.

### Control/Comparison Group

Participants in the control group completed the same study assessments (eg, parent surveys and daily diaries) as the intervention group. However, they did not receive any intervention materials until after the follow-up assessments were completed. Families in the control group received one FV box and all educational materials delivered at once via hard copies in early July 2025.

### Assessments

#### Parent Surveys

The parent survey assessed the following domains: parent and child diet [34], FI [35,36], nutrition security [13,37], parent feeding practices [38], child eating behaviors [39,40], as well as contextual factors that may influence the main outcomes of interest. These included parent mental health [41], parenting stress [42], household disorganization [43,44], parenting skills [45], competence [46], and satisfaction [47]. All survey measures are described in Table 2. On average, participants completed the parent survey in 36.35 (SD 16.74) minutes. This information was extracted from Qualtrics survey metadata, which includes duration in seconds. Parents were texted up to 3 reminders to complete their parent survey at each time point.

**Table 2.** Parent survey measures.

Domain and measure	Items, n
Child and parent diet	
National Cancer Institute’s Dietary Screener Questionnaire [34]	54
Food and nutrition security	
United States Department of Agriculture Household Food Security Survey Module [35]	22
Nutrition Security, Healthfulness Choice, Dietary Choice [13]	26
The Other 3 Pillars: Availability, Utilization, Stability [37]	16
Home Availability of Fruits and Vegetables Scale [48]	6
Parent feeding practices	
Child Feeding Questionnaire [38]	18
Child eating behaviors	
The Children’s Eating Behavior Questionnaire [39,40]	35
Child Food Preference Checklist [48]	58
Contextual factors	
Demographics	4
Parent Sense of Competency Scale [46]	17
Parenting and Family Adjustment Scale [45]	9
Confusion, Hubbub, and Order Scale [43,44]	15
Center for Epidemiologic Studies Short Depression Scale [41]	10
The Parental Stress Scale [42]	17
Kansas Parental Satisfaction Scale [47]	3
Rules and routines [49,50]	5

#### Daily Diaries

At each time point, parents completed electronic daily diaries for a 7-day period to assess relevant day-level behaviors/contextual factors. Using modified/abbreviated versions of the cited scales, parents reported on day-level parent and child diet [48], access to food [51], parent feeding practices [38], and child eating behaviors [39,40]. Daily diary items and survey flow are included in Table 3. Each daily diary took parents an average of 5.33 (SD 2.93) minutes to complete (survey duration included in Qualtrics metadata). Automated text messages were sent at 8 PM with a personalized link specific to that day as a reminder to complete the daily diary. Daily diaries expired after 24 hours so that parents were only able to complete one daily diary per day. Parents who completed at least 5/7 daily diaries were considered compliant with the study protocol. Up to an additional 7 days of daily diaries were sent to participants who requested additional survey opportunities.

**Table 3.** Daily diary items and survey flow.

Variable domain and survey item	Response options and logical flow
Child fruit and vegetable consumption	
Did you serve your child a fruit or vegetable today?	Yes Continue with fruit and vegetable consumption questions No Skip to parent feeding practices questions
What fruit or vegetable did you serve your child?	Free response
How much ________ did your child eat?	None Taste A little Some A lot
Did your child like ________?	Disliked a lot Disliked a little Neither disliked nor liked Liked a little Liked a lot
Did you serve your child another fruit or vegetable today?	Yes Repeat fruit and vegetable consumption questions No Skip to parent feeding practices questions Participants were shown this question up to 2 times.
Parent feeding practices [38]	
Today, I offered my child their favorite foods as a reward for good behavior.	Disagree Slightly disagree Neither agree nor disagree Slightly agree Agree
Today, I made sure that my child did not eat too many unhealthy foods.	Disagree Slightly disagree Neither agree nor disagree Slightly agree Agree
Today, I tried to get my child to eat when they were hungry.	Disagree Slightly disagree Neither agree nor disagree Slightly agree Agree
Food security status [35]	
Today, I worried whether our food would run out before we got money to buy more.	Not true Somewhat true True Don’t know
Today, the food we bought just did not last and we did not have money to get more.	Not true Somewhat true True Don’t know
Parent affect	
How .... did you feel today? [52] frustrated/angry sad/depressed stressed [53] happy calm/relaxed	Not at all Slightly Somewhat Moderately Extremely
How certain do you feel you can deal with all things that you have to do right now? [54]	Not at all Slightly Somewhat Moderately Extremely
Sleep quality	
How would you rate your sleep quality last night? [55]	Terrible Poor Average Good Excellent
How would you rate your child’s sleep quality last night? [56]	Terrible Poor Average Good Excellent
Parent sleep routines [57]	
What time did you go to bed last night?	Dropdown with time choices in 30-min increments from 7 PM to 8:30 AM None of these options are close to my bedtime. Fill in the blank with appropriate time.
What time did you wake up this morning?	Dropdown with time choices in 30-minute increments from 5 AM to noon None of these options are close to my wake time. Fill in the blank with appropriate time.
Child sleep routines [57]	
What time did your child go to bed last night?	Dropdown with time choices in 30-minute increments from 5 PM to 5:30 AM None of these options are close to my child’s bedtime. Fill in the blank with appropriate time.
What time did your child wake up this morning?	Dropdown with time choices in 30-min increments from 5 AM to noon None of these options are close to my child’s wake time. Fill in the blank with appropriate time.
Child nap [57]	
Did your child take a nap today?	Yes No Skip to rules and routines
What time did your child fall asleep for their first nap? What time did your child wake up from their first nap?	Dropdown with time choices in 30-min increments from 9 AM to 5 PM None of these options are close to when my child fell asleep for their nap. Fill in the blank with appropriate time.
Did your child take another nap today?	Yes No Participants were shown these questions up to two times.
Rules and routines [58]	
How much did you stick to your usual routines and rules for your child’s .... today? bedtime screen time	Not at all Slightly Somewhat Mostly Completely
Time with child [59]	
Since waking up this morning, how many hours have you spent with your child in the same location?	Dropdown with time choices in one-hour increments from less than 1 hour to 10 hours or more.
Daycare [58]	
How many hours did your child attend preschool/daycare today?	Dropdown with time choices in two-hour increments from not attending childcare today to attending for more than 12 hours.
Illness [58]	
Were you sick today?	Yes No
Was your child sick today?	Yes No
Child food and beverage consumption [60]	
Did your child consume ... today? low-fat or nonfat potato chips, tortilla chips, puffs, or corn chips regular potato chips, tortilla chips, puffs, or corn chips other salty snacks such as cheese nibs, Chex Mix, or Ritz Bitz any candy, including chocolate, Jelly Bellies, gummies, or life savers any doughnuts, Pop Tarts, or breakfast pastries any cookies, brownies, pies, or cakes low-fat or nonfat desserts such as low-fat ice cream or frozen yogurt any regular ice cream or milkshakes orange juice, apple juice, or any other 100% juice drink fruit drinks such as Snapple, Capri-Sun, or Kool-Aid sports drinks such as Gatorade or Powerade flavored waters such as Propel or vitamin-fortified waters diet soda or pop regular soda or pop any energy drinks such as Rockstar, Red Bull, Monster, or Throttle 1% or skim (nonfat) milk regular (whole) or 2% (reduced fat) milk any vegetables, including green salad, peas, green beans, or corn (not including fried potatoes or French fries) any fruit such as banana, apples, or grapes (do not include juice)	Yes No
Free response feedback	
Is there anything else you would like to tell us about your day?	Free response

#### Exit Survey

The exit survey was embedded in the posttest parent survey for those who received the Project V.E.G.G.I.E. intervention. It was designed to assess acceptability and utility of the FV boxes and educational materials via the following domains: quantity, variety, quality, consumption, waste, familiarity, enjoyment, helpfulness, and program impacts. See Table 4 for exit survey measures and questions.

**Table 4.** Exit survey measures [61].

Domain	Variable	Response options
Satisfaction	How satisfied were you with the ... amount of fruits in your boxes? amount of vegetables in your boxes? variety of fruits and vegetables in your boxes? quality of fruits and vegetables in your boxes?	Very dissatisfied Dissatisfied Neither satisfied nor dissatisfied Satisfied Very satisfied
Consumption	How much of the fruits and vegetables from your boxes were eaten by ... adults in your home? children in your home? people who do not live in your home?	None A little Some A lot Most
Waste	How much of the fruits and vegetables from your boxes were not eaten by anyone?	None A little Some A lot Most
Familiarity	How familiar were you with the items in your fruit and veggie boxes?	Very familiar Moderately familiar Somewhat familiar Slightly familiar Not at all familiar
Enjoyment	How much did your family like the items in your fruit and veggie boxes?	Strongly like Like Neither like nor dislike Dislike Strongly dislike
Helpful	How helpful were the boxes for feeding your family?	Very helpful Moderately helpful Somewhat helpful Slightly helpful Not at all helpful
Education utility	How useful was the advice included with your fruit and veggie boxes?	Very useful Moderately useful Somewhat useful Slightly useful Not at all useful
Education acceptability	How much did you like the advice included with your fruit and veggie, boxes?	Strongly like Like Neither like nor dislike Dislike Strongly dislike
Impact on adult consumption	Did the boxes allow you to eat more fruits and vegetables?	Not at all A little Some More Many more
Impact on child consumption	Did the boxes allow your child to eat more fruits and vegetables?	Not at all A little Some More Many more
Impact on purchasing	While receiving the boxes, how has your purchasing of fruits and vegetables changed?	Decreased a lot Decreased somewhat Stayed the same Increased somewhat Increased a lot
Recommendation	Would you recommend this program to others with preschool-age children?	Definitely not Probably not Maybe Probably yes Definitely yes
Free response feedback	Is there anything else you would like to tell us about the Project V.E.G.G.I.E.^a^ program?	Free response

a V.E.G.G.I.E.: Vegetable Eating Gets Going by Increasing Exposure.

#### Exit Interviews

Both intervention and control participants were invited via text message to complete exit interviews about their experience with the study. Exit interviews were conducted in the participant’s preferred language (either English or Spanish) over the phone using a semistructured interview guide. Interviews took approximately 30 minutes, and questions assessed participants’ satisfaction with the Project V.E.G.G.I.E. program, including the ease of picking up the FV boxes at the childcare centers, the topics covered in the educational materials, as well as the quality and quantity of produce in the boxes. Participants in the control group were asked questions about their satisfaction with the study protocol and their one box of FVs. The semistructured interview guides for both groups are included as Multimedia Appendices 1 and 2. All exit interviews were recorded and transcribed using Otter.ai, an online transcription software. Transcriptions were imported into NVivo (version 15.3; Lumivero) software for data analysis, conducted by trained research assistants using an immersion crystallization approach (inductive) and constant comparison methodology.

#### Compensation

Parents received up to US $175 in Amazon e-gift card codes via text message. Gift card codes were distributed after completion of each assessment. Table 5 presents participant compensation by assessment and time point.

**Table 5.** Participant compensation by assessment and time point^a^.

Time point and assessment	Compensation
Baseline	
Parent survey	US $25
Daily diaries	US $25
Posttest	
Parent survey	US $25
Daily diaries	US $25
Exit interviews: intervention group	US $25
Follow-up	
Parent survey	US $25
Daily diaries	US $25
Exit interviews: control group	US $25

a Participants received US $25 for completing each assessment at each time point for a total of US $175 in Amazon e-gift card codes.

### Statistical Analysis

#### Feasibility

Descriptive statistics of the feasibility outcomes will be presented as: recruitment (number of families who complete the screener), enrollment rate (proportion of families enrolled relative to the number of eligible families), retention rate (proportion of families who remain in the study across time points relative to the total number of participants), and adherence to the study protocol/missing data (proportion of parent surveys and daily diaries completed at each time point). We will evaluate intervention fidelity as delivery of FV boxes and educational materials. Delivery of FV boxes and printed educational materials will be directly assessed by onsite undergraduate research assistants. Educational materials sent via text message will be documented by a trained graduate research assistant. Participant barriers and facilitators to receiving the intervention (FV boxes; education) will be included in the exit interviews. In line with previous pilot/feasibility studies among similar populations (families with low resources), the protocol and intervention will be deemed feasible if enrollment, retention, and protocol compliance rates of at least 70% are achieved [58,61-64].

#### Acceptability

The proportion of participants who indicate they are satisfied with each facet of the program will be reported; the program will be considered acceptable to families if at least 70% of participants indicate they are satisfied/very satisfied across measures.

#### Progression Criteria

Progression to larger-scale trials will be determined jointly with community partners based on feasibility and acceptability metrics. It is expected that the intervention will be refined and the protocol modified prior to future iterations of the Project V.E.G.G.I.E. study in order to enhance recruitment, retention, acceptability, and intervention fidelity.

#### Efficacy

To assess an initial signal of program efficacy, we will assess whether those who received the intervention changed differently than those in the control group over time (time-by-treatment interaction). We will conduct a series of mixed-effects models, accounting for the nesting of repeated measures within participants with one analysis per outcome of interest: diet, FI, nutrition security, parent feeding practices, and child eating behaviors. Full information maximum likelihood estimation will be used to account for missing data and allow all available data to be included in the models. Standard effect size estimates (Cohen *d*) will be calculated as baseline to posttest and baseline to follow-up. Results will be interpreted with caution due to small sample size and limited statistical power. Due to the small number of childcare providers (n=5), analyses will not account for nesting of participants within sites. Demographic characteristics will be presented separately for those in the treatment and control conditions, and differences will be evaluated (independent samples *t* tests). We will consider and discuss detected differences between groups and their potential impacts on study outcomes. Analyses will be conducted in Stata 18.0 statistical software (StataCorp LLC).

### Sample Size

This pilot/feasibility study consisted of a sample size of 20 participants recruited from 5 childcare providers. This sample size is sufficient to provide an initial signal of the feasibility and acceptability of the Project V.E.G.G.I.E. intervention [65]. A well-powered RCT is needed to evaluate study outcomes; as such, a power analysis was not necessary for this pilot/feasibility nonrandomized trial.

## Results

The Project V.E.G.G.I.E. Pilot/Feasibility Study was internally funded by the Department of Health Education and Behavior in the College of Health and Human Performance at the University of Florida. Recruitment of participants (n=20) began in February and ended in March 2025. The study protocol and data collection took place from March to July 2025. Data cleaning and analyses are underway (January 2026), and the outcome paper for this pilot/feasibility study is expected to be submitted for publication in fall 2026.

## Discussion

### Overview

Ultimately, Project V.E.G.G.I.E. aims to increase FV consumption among preschool-age children and their families at risk of experiencing FI through enhanced nutrition security (increased FV access) and education tailored to the needs of parents of preschoolers. The underlying premise of Project V.E.G.G.I.E. is that children from families at risk of experiencing FI lack the necessary exposure to FVs, an unexplored factor contributing to inadequate FV consumption. Our study protocol overcomes the weaknesses of previous interventions using increased FV access. Programs that provide individuals with reduced- or no-cost FVs often involve many challenges, such as advanced purchasing/ordering and inconvenient pick-up times/locations. We aim to create a more convenient program by delivering FVs to childcare providers, an ideal time and location for parents of preschool-age children, thus increasing program uptake and use. In addition to increased access, we provided parents with educational materials focused on evidence-based parent feeding practices aimed at increasing child FV consumption. By targeting both FV access and parent feeding practices, we hypothesize that Project V.E.G.G.I.E. will result in improved program acceptability, treatment fidelity, and ultimately a more efficacious approach to increasing FV consumption among preschool-age children at risk of experiencing FI.

### Strengths and Limitations

A key strength of this research study is the capable and experienced community partners who assisted with participant recruitment and intervention implementation: ELCOC and Second Harvest Food Bank of Central Florida. By leveraging the existing ELCOC network of childcare providers serving families with low resources, we were able to connect with 5 childcare providers eager to be involved with the pilot/feasibility study, as demonstrated by our 100% recruitment rate. Their large network (632 childcare centers and 31,761 children) [66] will enable us to recruit additional sites/participants for future iterations of the Project V.E.G.G.I.E. intervention.

The Second Harvest Food Bank of Central Florida is a private, nonprofit organization that has served the community for over 40 years. They collect, store, and distribute food to more than 750 feeding partners in seven Central Florida counties. In 2024, they distributed enough food for 82 million meals to partner programs [67]. Their “Bring Hope Home” initiative delivers boxes of food (including fresh FVs) to the homes of senior citizens and individuals with disabilities [68,69]. As such, Second Harvest was able to leverage this existing infrastructure to build the FV boxes for Project V.E.G.G.I.E. They have the resources and expertise to support Project V.E.G.G.I.E. as we scale up to a well-powered cluster RCT and provide support to additional families throughout central Florida.

This study is not without limitations. As a pilot/feasibility study, we are not adequately powered to evaluate intervention impacts or account for clustering within childcare providers. As such, between-group differences may be due to differences at the site level that cannot be disentangled from the intervention effects, limiting our ability to determine causation. A well-powered cluster RCT is necessary to evaluate the efficacy of the Project V.E.G.G.I.E. intervention. Because the intervention is implemented at childcare providers, it is necessary for all participants enrolled at a given site to receive the same treatment. This study design reduces the risk of contamination and is essential for our community partners. It would be unethical to supply food to only some families while withholding it from others who witness its distribution.

Our sample (n=5 childcare providers; n=20 parent/child dyads) recruited from Orange County, Florida, is not generalizable to the entire population of US families at risk of experiencing FI. However, there are a number of reasons why Central Florida is an ideal location to conduct this work. Orange County, Florida, is racially/ethnically diverse, with approximately 37% of the population categorized as Non-Hispanic White, 19% Non-Hispanic Black, and 33% Hispanic [70]. In 2023, 23% of residents were foreign born [70], and 13% of residents and 15% of residents with children were classified as below the Federal Poverty Line, slightly higher than the national average [70]. Further, although the Orlando area is classified as urban/suburban, the county also includes “Rural Residential Enclaves,” single-family neighborhoods with low residential densities similar to rural settings. Thus, this location will enable future Project V.E.G.G.I.E. studies to assess intervention efficacy and unique barriers/facilitators across varying levels of urbanicity/rurality.

As previously mentioned, the control group was not initially included in the study. It was added later due to potential changes in federal policy that could impact the target population. Because of this late addition, recruitment and baseline dates differed for the intervention and control, resulting in potential site-selection bias and temporal confounding.

Due to limited funding, this pilot/feasibility study relies on self/parent-report to evaluate all outcomes of interest, including diet, food and nutrition security, parent feeding practices, and child eating behaviors. Although there is error inherent in self/parent-reported measures, we attempt to improve data quality by collecting day-level data (improved recall) and using validated measures. In the future, we hope to address these weaknesses by conducting a well-powered cluster RCT that includes an objective measure of FV consumption (eg, VeggieMeter) [71-73].

### Conclusions

This study will provide insight into the feasibility and acceptability of the intervention and study protocol. Feedback from participants will enable us to refine the Project V.E.G.G.I.E. intervention prior to scaling up to a well-powered cluster RCT. Ultimately, policy and research implications of this work include facilitating programs that provide families with preschool-age children with increased access to FVs, as well as education around evidence-based parent feeding practices. Integrating such programs into childcare settings may overcome barriers typically associated with initiatives to increase FV access.

## Supplementary material

10.2196/93866 Multimedia Appendix 1 Exit interview guide for intervention participants.

10.2196/93866 Multimedia Appendix 2 Exit interview guide for control participants.

## Abbreviations

ELCOC: Early Learning Coalition of Orange County
FI: food insecurity
FV: fruit and vegetable
RCT: randomized controlled trial
V.E.G.G.I.E.: Vegetable Eating Gets Going by Increasing Exposure
