# Supplementary material for: Increasing Fruit and Vegetable Consumption Among Preschool-Age Children and Their Families: Protocol for the Project V.E.G.G.I.E. Pilot/Feasibility Study
Source: JMIR Res Protoc. 2026 Nov 13;15:e93866. doi: 10.2196/93866 (PMC13766664; doi:10.2196/93866)
Supplement: Supplementary file 1 [file resprot-v15-e93866-s001.docx]

# **Exit Interviews- Intervention**

Hi {participant name}. I am ___________________, a researcher at the University of Florida. Thank you so much for talking with me about the Project VEGGIE program. Your input will be so valuable to us as we make changes to the program for future participants.

We will discuss your thoughts on the fruit and veggie boxes, and the educational content. For these interview questions, there are no right or wrong answers. We want to learn about what worked and didn’t work for your family. Your personal experiences and perspective are important to us.

Before we get started, I want to remind you that since you indicated you were okay with recordings, we will be recording this interview. This is so that we can analyse the data later. Do you have any questions before we get started?

**Interview Questions**

**Let’s begin by talking about the fruit and veggie boxes.**

Tell me your thoughts about the fruit and veggie boxes.

a. What did you like about them?
b. Was there anything that could have been improved?
c. What did you think about the quality of the produce in the boxes?
d. What did you think about the amount of the produce in the boxes?
e. What did you think about receiving the box at your child’s childcare center?

I see that you were able to pick up ____ of your 6 boxes. For the boxes you missed, what prevented you from receiving the boxes?

**Now let’s talk about educational materials.**

Tell me your thoughts about the educational materials.

a. What did you like about them?
b. Was there anything that could have been improved?
c. What did you think about the topics that were covered?
d. What is missing from the educational materials? Is there anything you would like more information on that was not included?
e. Which strategies did you try?
f. Was there anything you tried that worked particularly well?
   i. If so, tell me about it.
g. Was there anything you tried that did not go well?
   i. If so, tell me about it.
h. Was there anything suggested that you did not try?
   i. If so, why not?

***If someone does not remember the educational materials, you can remind them that there were handouts with topics like:

- not pressuring children to try new foods
- serving the foods you want them to try over and over
- getting kids involved in preparing foods
- Not sneaking foods

**Let’s talk about the program overall**

Tell me about how the program impacted your family?

a. Did you make any changes because of the program?
   i. If so, tell me about them
b. Did your family eat more fruits and vegetables because of the program?
   i. What about your child?
c. Did your family try any new fruits or vegetables because of the program?
   i. What about your child?
d. What would you tell a friend about Project VEGGIE?
e. Is there anything else you would like to tell us?
