# Supplementary material for: Increasing Fruit and Vegetable Consumption Among Preschool-Age Children and Their Families: Protocol for the Project V.E.G.G.I.E. Pilot/Feasibility Study
Source: JMIR Res Protoc. 2026 Nov 13;15:e93866. doi: 10.2196/93866 (PMC13766664; doi:10.2196/93866)
Supplement: Supplementary file 2 [file resprot-v15-e93866-s002.docx]

# **Exit Interviews- Control Group**

Hi {participant name}. I am {your name}, a researcher at the University of Florida. Thank you so much for talking with me about the Project VEGGIE program. Your input will be so valuable to us as we make changes to the program for future participants.

We will discuss your thoughts on the study protocol, the fruit and veggie box, and the educational content. For these interview questions, there are no right or wrong answers. We want to learn about what worked and didn’t work for your family. Your personal experiences and perspective are important to us.

Before we get started, I want to remind you that since you indicated you were okay with recordings, we will be recording this interview. This is so that we can analyse the data later. Do you have any questions before we get started?

**Interview Questions**

**Let’s begin by talking about the Project VEGGIE parent surveys. These are the longer surveys.**

**For people who completed at least one parent survey:**

a. What did you think about completing these surveys?
b. Were there any questions that you were not expecting?
   - If so, which questions.
c. Were there any questions that you did not understand?
   - If so, which questions.
d. Were there any questions that you did not like or did not feel comfortable answering?
   - If so, which questions.
e. What did you think about the LENGTH of the parent survey?
   - About how long do you think the surveys took you to complete. We have total time from start to finish, but if someone takes a break after starting, we aren’t able to tell.
f. ONLY FOR PEOPLE WHO MISSED ONE OF THE SURVEYS. I see that you were able to complete ____ of the 3 parent surveys. Thanks so much for doing this! For the one(s) you missed what happened?
   - Potential prompt: Is there anything we could do to help people complete these surveys: more reminders, reminders at different times, sent in a different way?
g. What did you think about receiving $25 gift cards for completing these surveys? Did this amount seem right to you?
   - Do you think you would complete more surveys if the gift card amount was higher? What seems like a good amount to you considering how long these surveys take?
h. Is there anything else you would like to tell us about the parent surveys?

For people who did not complete ANY parent surveys:

a. I see that you were not able to complete any parent surveys. Is there anything we could do to help people complete these surveys

- Potential prompts. Would more reminders be helpful? What about reminders at different times? Would it be better to send the surveys in a different way?

b. What did you think about receiving $25 gift cards for completing these surveys?
   a. Do you think you would have completed more surveys if the gift card amount was higher? What seems like a good amount to you?

**Now, let’s talk about the daily diaries. These are the short surveys that were sent out to you each night.**

a. What did you think about completing the daily diaries?
b. Were there any questions that you were not expecting?
   - If so, which questions.
c. Were there any questions that you did not understand?
   - If so, which questions.
d. Were there any questions that you did not like or did not feel comfortable answering?
   - If so, which questions.
e. What did you think about the LENGTH of the daily diaries?
f. What did you think about the TIMING of the daily diaries? They were sent out at 8PM.
   - Is there another time that would work better for you?
g. I see that across the three time points you were able to complete ________ daily diaries. Thank you so much for doing this! On the nights when you did not complete the daily diary, what happened?
   - Is there anything we could do to help people complete the daily diaries?
h. What did you think about receiving $25 gift cards for completing each week of daily diaries?
   - Did this amount seem right to you?
   - Do you think you would have completed more surveys if the gift card amount was higher? What seems like a good amount to you?
i. Is there anything else you would like to tell us about the daily diaries?

**Let’s talk about the fruit and veggie box. Your family should have received one box.**

a. Tell me your thoughts about it.

- What did you like about it?
- Was there anything that could have been improved?
- What did you think about the quality of the produce in the box?
- What did you think about the amount of the produce in the box?

b. What did you think about receiving the box at your child’s childcare center?

**Now let’s talk about educational materials.**

Tell me your thoughts about the educational materials.

a. What did you like about them?
b. Was there anything that could have been improved?
c. What did you think about the topics that were covered?
d. What is missing from the educational materials? Is there anything you would like more information on that was not included?
e. Which strategies did you try?
f. Was there anything you tried that worked particularly well?
   i. If so, tell me about it.
g. Was there anything you tried that did not go well?
   i. If so, tell me about it.
h. Was there anything suggested that you did not try
   i. If so why not?

**Let’s talk about the program overall**

a. What would you tell a friend about Project VEGGIE?
b. Is there anything else you would like to tell us?
